# Chlorogenic acid reduces inflammation in murine model of acute pancreatitis

**DOI:** 10.1007/s43440-021-00320-5

**Published:** 2021-08-12

**Authors:** Aleksandra Tarasiuk, Kamila Bulak, Marcin Talar, Jakub Fichna

**Affiliations:** 1 Department of Biochemistry, Faculty of Medicine, Medical University of Lodz, Lodz, Poland; 2 Sub-Department of Pathomorphology and Forensic Veterinary Medicine, Department and Clinic of Animal Internal Diseases, Faculty of Veterinary Medicine, University of Life Sciences in Lublin, Lublin, Poland

**Keywords:** Acute pancreatitis, L-arginine induced acute pancreatitis murine model, Chlorogenic acid, Immunohistochemistry, Myeloperoxidase activity, Inflammation

## Abstract

**Background:**

The pathogenesis of acute pancreatitis (AP) initiation and progression is still unknown, and effective treatment is limited to supportive care. Many phytochemicals have the potential to alleviate AP symptoms and may be a useful and effective supplement to standard AP treatment. The objective of the study was to examine the potential role of chlorogenic acid (CGA), a polyphenol known for anti-inflammatory effect, in the treatment of experimental AP in mice.

**Methods:**

Two intraperitoneal (*ip*) injections of L-arginine (dosage 400 mg/100 g BW) were given 1 h apart to generate the AP murine model. Mice were separated into two experimental groups after 12 h from the first L-arginine injection: AP mice treated with CGA (oral gavage (*po*) every 12 h; 20 mg/kg BW) and non-treated AP mice (*po* vehicle, 5% dimethyl sulfoxide every 12 h). Every 12 h, control mice were given an equivalent volume of vehicle. At 72 h, mice were slaughtered. Histology, as well as myeloperoxidase (MPO) and amylase activity assays, were performed on pancreatic tissues.

**Results:**

In murine mouse model of AP *po* administration of CGA decreased MPO vs. AP (40.40 ± 2.10 U vs. 7.39 ± 0.34*; p* < *0.001*) as well as amylase activity vs. AP (1444 ± 56 mU/mL vs. 3340 ± 144 mU/mL, Fig. 2B; *p* < *0.001*). When comparing CGA mice to AP mice, histological research demonstrated that the severity of AP was reduced following CGA treatment.

**Conclusions:**

The current study found that CGA might have anti-inflammatory effect on L-arginine-induced pancreatitis. Dietary intervention with CGA may be advised as a supportive treatment for AP, according to our findings.

**Graphic abstract:**

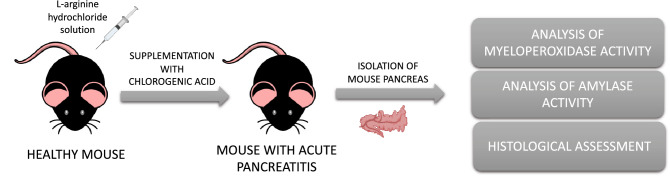

## Introduction

Acute pancreatitis (AP) is a disorder of the mechanisms that inhibit or stabilize the activity of enzymes in the follicular cells of the exocrine pancreas, which in turn causes the activation of proteolytic and lipolytic pancreatic enzymes. The result can be complete destruction of the pancreas, or internal hemorrhage, caused by the digestion of the walls of the blood vessels and the gastrointestinal tract by active enzymes. In most patients, the disease is mild. However, in about 20–30% cases there are fatal consequences. The patient's death occurs very early, even a week after the onset of the first symptoms, due to a multi-organ failure. Death may also occur from sepsis around the third week of the disease; sepsis leads to an inflammatory reaction and thus an infection that may result in organ failure or dysfunction [1–5].

The diagnosis of AP is most often established by the presence of two of the three following criteria: (i) abdominal pain consistent with the disease, (ii) laboratory tests (serum amylase and/or lipase activity greater than two/three times the upper limit of normal), and/or (iii) characteristic findings from abdominal imaging [6].

Treatment of milder AP is relatively simple and involves short-term fasting (3–4 days) accompanied by intravenous rehydration and administration of painkillers. All other cases of AP are treated in the hospital, where patients are closely monitored for signs of serious problems and given supportive treatment, such as fluids and oxygen [1–4, 7–9].

**Fig. 1:**
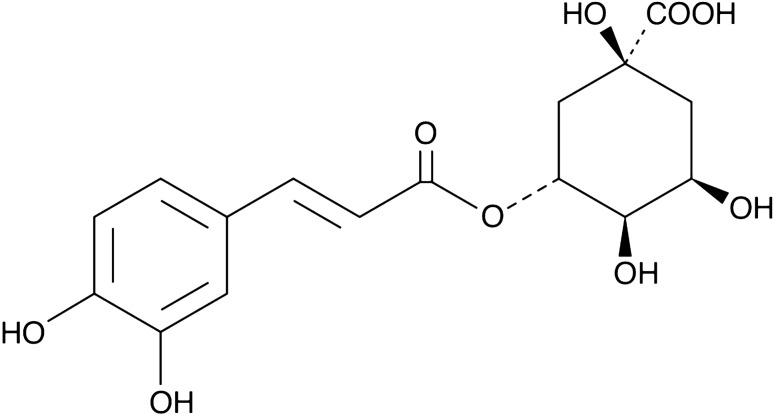
Chemical structure of chlorogenic acid (5-caffeoylquinic acid, CGA), which is formed via the esterification of caffeic acid and quinic acid

Chlorogenic acid (CGA) is a polyphenol that is an ester of caffeic acid and quinic acid (Fig. 1). It is primarily found in green (unroasted) coffee beans, cocoa leaves and seeds, yerba mate (dried, ground leaves of a native species of holly tree, *Ilex paraguariensis*), hawthorn, yam, artichoke, blueberry, chokeberry and mulberry, tomatoes, raw potatoes, but also in honey and some herbs [10–13]. According to the findings of some studies, regular consumption of green coffee may have antibacterial, anti-inflammatory, and anti-cancer properties. [14, 15]. CGA can also help to reduce the absorption of carbohydrates from food and lower blood pressure [16, 17]. Furthermore, it has been proposed that CGA may improve brain function and have neuroprotective effects on its own [18]. Contrastingly, the results of many studies present that CGA has an anti-inflammatory effect. This compound is said to decrease the inflammatory response by indirectly inhibiting the expression of pro-inflammatory cytokines (interleukin (IL)-1β, IL-6, tumor necrosis factor α (TNF-α)) and chemokines (including chemokine (C-X-C motif) ligand 1, IL-8) [19]. CGA also reduces the concentration of prostaglandins, which are inflammation mediators, and nitric oxide, which reacts with free radicals to form compounds that can damage DNA and cause mutations by inhibiting cyclooxygenase-2 (COX-2) [10, 12, 13, 15, 19].

Even though the beneficial role of CGA is well-explored in many inflammatory diseases [10], the effects of CGA on established AP have not been examined. In this study, we tested the anti-inflammatory effect of CGA using the mouse model of L-arginine-induced AP which is well known as a mimic of clinical pancreatitis and is commonly used to evaluate the effects of phytochemicals [20–22].

## Materials and methods

### Animals

Male C57BL/6 mice, 8–12 weeks of age, were obtained from the Animal House at the University of Lodz, Lodz, Poland. All animals were housed in standard rodent cages in a climate-controlled room with an ambient temperature of 23 ± 2 °C and 12:12 h light–dark cycle. Animals were fed standard laboratory chow, given water ad libitum, and randomly assigned to control or experimental groups. All animal protocols were approved by the Medical University of Lodz Animal Care Committee (#70/LB83/2017). All efforts were made to minimize animal suffering and to reduce the number of animals used.

Animals were divided into three experimental groups (*n *= 8):

1. Control
2. Mice with acute pancreatitis (AP)
3. Mice with acute pancreatitis treated with CGA (AP + CGA)

### Induction of acute pancreatitis

A sterile solution of L-arginine hydrochloride (8%) was prepared in normal saline, and the pH was adjusted to 7.0. The arginine solution was administered intraperitoneally (*ip*) to nonfasted mice at a dose of 4 g/kg BW. Animals were then returned to the cages and allowed free access to food and water. After 1 h, animals were administered a second dose of L-arginine hydrochloride solution (4 g/kg BW) in saline. Controls received each time a sham injection of saline alone. Mice were weighed daily and monitored for clinical symptoms of pancreatitis [23].

### Pharmacological treatment

CGA was administered two times daily at the dose of 20 mg/kg orally (*po*, 150 μL) with the first treatment 12 h after induction of L-arginine acute pancreatitis mouse model. CGA was dissolved in 5% dimethyl sulfoxide (DMSO) in saline, which was used as vehicle. Control and AP mice received equivalent volume of vehicle every 12 h. Mice were sacrificed at 72 h. Pancreatic tissues were collected for histology in 4% buffered formalin as well as snap frozen in liquid nitrogen for myeloperoxidase (MPO) and amylase activity analysis.

### Determination of tissue myeloperoxidase (MPO) activity

To monitor the degree of inflammation, MPO activity assay was performed as described earlier [24]. Following isolation, isolated pancreas sections were weighed and homogenized in hexadecyltrimethylammonium bromide (HTAB) buffer (final concentration—50 mg tissue/1 mL HTAB buffer) using a Precellys Evolution advanced tissue homogenizer (Bertin Instruments, France). Following centrifugation (13,200 g, 15 min, 4 °C), supernatants were collected from each Eppendorf tube and transferred to another Eppendorf tube. Further, 7 μL of supernatant was added on each well on a 96-well plate, containing 200 μL of 50 mM potassium phosphate buffer (pH 6.0), 0.167 mg/mL of O-dianisidine hydrochloride, and 0.05 μL of 1% H_2_O_2_. Absorbance was measured in triplicate at optical density (OD) 450 nm after 30 and 60 s (iMARK Microplate Reader, Biorad, UK). MPO was measured in milliunits per gram of wet tissue, with 1 unit equaling the amount of enzyme capable of converting 1 mol of H_2_O_2_ to water in 1 min at room temperature. MPO activity was measured in units (U) per minute using a standard curve and purified peroxidase enzyme.

### Determination of amylase activity in the pancreas

Amylase Assay Kit (Colorimetric) (Abcam, ab102523) was used to detect activity of *α*-amylase through a two-step reaction, according to manufacturer’s protocol.

### Histology

Pancreas tissue samples were fixed for 24 h in 4% buffered formalin, pH 7.2, and processed automatically in a tissue processor (Leica TP-1020). Paraffin-embedded tissue samples were sectioned, mounted on slides, and stained with hematoxylin and eosin (H&E); concurrently, Masson’s trichrome staining and immunohistochemical reaction (IHC) were performed (see details below). Samples were examined using Nikon’s Eclipse E600 light microscope (Nikon Instruments Inc., Japan). Photographs were taken using a digital imaging system consisting of a digital camera for microscopy (Nikon DS-Fi1, Nikon Instruments Inc., Japan) and image analysis software (NIS-Elements BR-2.20, Laboratory Imaging, Czech Republic). Morphological analyses were done by a pathologist blinded to the experimental protocol.

#### H&E staining

Pancreas sections were stained with H&E staining in accordance with standard protocol [25].

#### Masson’s trichrome staining

The Masson’s trichrome staining was conducted using standard protocol [26].

#### IHC

The immunohistochemical reaction was performed based on the indirect immunoperoxidase method using a detection system (UltraVision Quanto Detection System HRP, ThermoScientific) and chromogen (DAB Quanto, ThermoScientific). Anti-Collagen 1 polyclonal antibody (ab21286, Abcam) at a dilution of 1:250 and anti-matrix metalloproteinase 1 (MMP-1) polyclonal antibody (ab137332, Abcam) at a dilution of 1:500 were used. Incubation with primary antibodies in the humid chamber lasted 60 min at room temperature. To block endogenous peroxidase, 3% hydrogen peroxide solution with methanol was used. Reaction sites were unmasked in a water bath at pH 6 and 95 °C to expose the epitopes. 2.5% horse serum solution (Normal Diluted Horse Serum, Vector Laboratories, USA) was used to block non-specific reaction sites. A tris-buffered saline (TBS, Sigma–Aldrich) solution was used instead of the original primary antibody to obtain negative control of the immunohistochemical assay. A hematoxylin solution (Novocastra, Leica Biosystems, USA) was used for counterstain of nuclei.

### Drugs

All drugs and reagents, unless otherwise stated, were purchased from Sigma–Aldrich (Poznan, Poland). CGA (purity 98% by high-performance liquid chromatography) was extracted from honeysuckle flowers and purchased from Nanjing Zelang Medical Technology Co. (Nanjing, Jiangsu, China). In all in vivo tests, drugs were dissolved in 5% DMSO in saline, which was used as vehicle in control groups. Vehicle affected none of the measured parameters when given alone.

### Statistical analysis

Statistical analysis was performed using Prism 9.0.1 (GraphPad Software Inc., La Jolla, CA, USA). The data are expressed as means ± SEM. The Shapiro–Wilk test was used to test the normality of data distribution. One-way ANOVA followed by Bonferroni post hoc test as well as student *t* test were used for analysis. *P* value < 0.05 were considered as statistically significant.

## Results

### Administration of CGA attenuated pancreatitis in L-arginine-induced AP in mice

Development of AP is accompanied by sequestration of neutrophils in the pancreas. Therefore, MPO activity in those tissues, which is a standard measurement of neutrophil infiltration, has been used as a biochemical marker of pancreatitis.

**Fig. 2:**
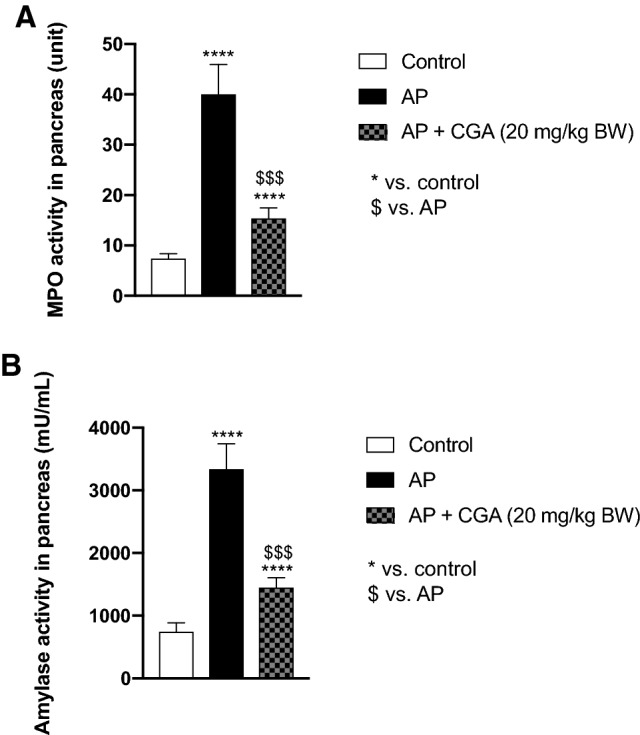
Myeloperoxidase (MPO) activity (**A**) and amylase activity (**B**) in the pancreas in the control group, acute pancreatitis (AP) group and following administration of chlorogenic acid (CGA) after 72 h from the induction of AP. One-way analysis of variance (ANOVA) followed by Bonferroni correction test was used to evaluate the variables between groups. Bars represent mean ± SEM of *n *= 8 mice per group; ****p < 0.0001 as compared with control. ^$$$^*p *< 0.001 vs. AP

Mice treated only with L-arginine had highly significantly increased MPO activity in the pancreas after 72 h compared with the control group (40.40 ± 2.10 U vs. 7.39 ± 0.34, Fig. 2A.; *p* = *0.000000000000081*). Administration of CGA (*po*, 20 mg/kg BW) highly significantly reduced MPO activity in the pancreas (15.40 ± 0.74 U vs. 40.20 ± 2.10 U, Fig. 2A; *p* = *0.000000000009161*) in the murine model of AP.

### Administration of CGA significantly reduced amylase activity in the pancreas in L-arginine-induced AP in mice

Elevated plasma amylase is an important marker of pancreatic acinar cell injury [6, 27]. Administration of L-arginine increased amylase activity in the pancreas compared to controls (3340 ± 144 mU/mL vs. 745 ± 49 mU/mL, Fig. 2B; *p* = *0.000000071764227*). Administration of CGA significantly suppressed the increase of the amylase activity in mice with L-arginine induced AP compared with the AP group (1444 ± 56 mU/mL vs. 3340 ± 144 mU/mL, Fig. 2B; *p* = *0.000001245393492*).

### Histological analysis revealed decreased severity of pancreatitis after CGA treatment in AP mice

#### H&E staining (Fig. 3A–C)

**Fig. 3:**
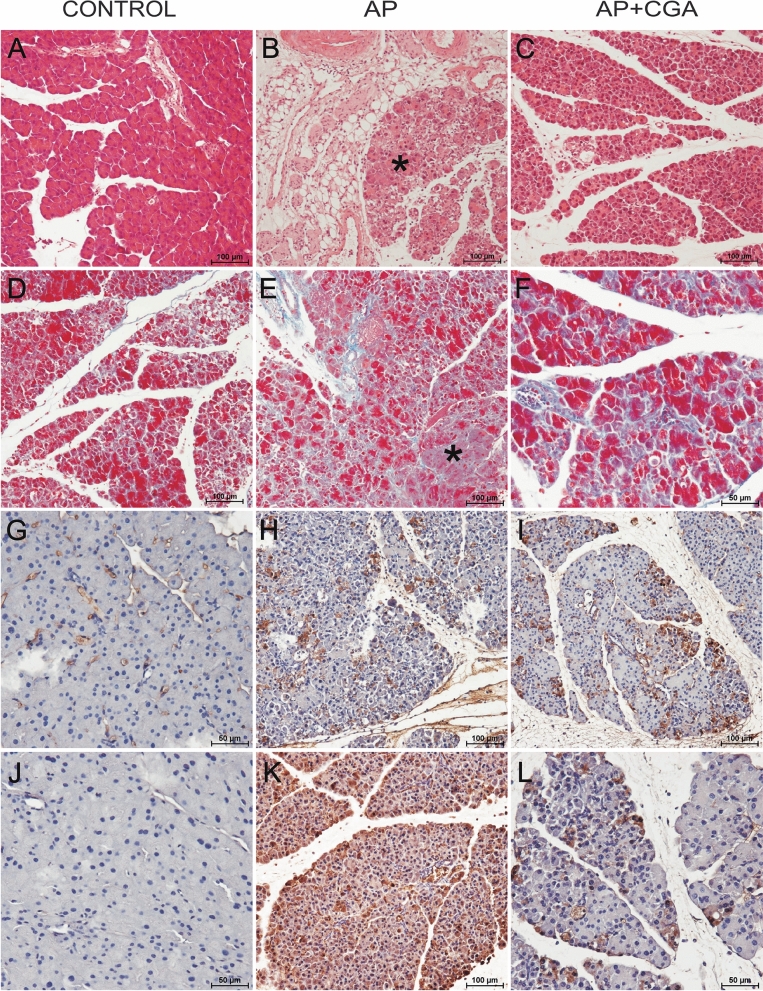
Representative microphotographs of the pancreas. H&E staining: control group (A)—normal architecture of the pancreas; AP group (B)—inflammatory infiltrate in adjacent fatty tissue and distortion of acini (star); AP + CGA group (C)—minimal inflammatory infiltrate in periacinar area and lack of atrophied acinar cells. Masson’s trichrome staining: control group (D)—minimal deposition of collagen fibers in periacinar area; AP group (E)—moderate collagen deposition in periacinar area and distinct atrophied acinar cells (star); AP + CGA group (F)—increased collagen fibers deposition between individual acinar cells. IHC/anti-Coll-1: control group (G)—weak reaction between individual acinar cells, AP group (H) and AP + CGA group (I)—heterogenous distribution of moderate to strong immunohistochemical reaction. IHC/anti-MMP-1: control group (J)—negative reaction; AP group (K)—strong positive reaction; AP + CGA (L)—multifocal weak to moderate reaction. Samples were examined using Nikon’s Eclipse E600 light microscope (Nikon Instruments Inc., Japan). Photographs were taken using a digital imaging system consisting of a digital camera for microscopy (Nikon DS-Fi1, Nikon Instruments Inc., Japan) and image analysis software (NIS-Elements BR-2.20, Laboratory Imaging, Czech Republic)

Histopathological evaluation of the H&E-stained pancreas tissue sections from the L-arginine treated group revealed significant acinar cells dissociation, disruption of histoarchitecture, acinar cell vacuolization and edema, moderate acinar cells atrophy, and neutrophil infiltration (Fig. 3B). In contrast, no microscopical changes were observed in the control group (Fig. 3A). There was minimal inflammatory infiltrate in periacinar area and lack of atrophied acinar cells in the AP group treated with CGA (Fig. 3C).

#### Masson’s trichrome staining (Fig. 3D–F)

Masson’s trichrome staining is used for the detection of collagen fibers in the pancreas. The overall amount of collagen deposited by fibroblasts in the extracellular matrix (ECM) is a regulated balance between collagen synthesis and collagen catabolism, which is a carefully controlled process in the pancreas.

In the control group there was a minimal deposition of collagen fibers (Fig. 3D), while in the AP group there was a moderate collagen deposition in periacinar area (Fig. 3E). After treatment with CGA, collagen fibers deposition was observed only between individual acinar cells (Fig. 3F).

#### IHC/anti-collagen 1 (anti-coll-1) (Fig. 3G–I)

As collagen fibers deposition is important in the remodeling of the ECM of the pancreas and its severity is the hallmark of chronic pancreatitis, to confirm results from Masson’s trichrome staining the IHC/anti-coll-1 reaction was performed. Collagen type I appeared as loose fibers surrounding acinar units and pancreatic islets. In the control group (Fig. 3G) there was a weak reaction between individual acinar cells, while in the AP group (Fig. 3H) and in the AP group treated with CGA (Fig. 3I) there was a heterogenous moderate to strong immunohistochemical reaction.

#### IHC/anti-MMP-1 (Fig. 3J–L)

Matrix metalloproteinases (MMPs), which belong to the family of zinc-dependent endopeptidases, degrade most of the constituents of the ECM, basement membrane, as well as inflammatory mediators. Most of the MMPs are not expressed in the normal tissue, but their expression and activity increase dramatically during wound healing as a result of a change in ECM composition, inflammation, and repair [28].

In the control group MMP-1 protein was not detectable (Fig. 3J), while AP group showed strong expression of MMP-1 (Fig. 3K). In the AP group treated with CGA there was a multifocal weak to moderate immunoreactivity for MMP-1 (Fig. 3L).

## Discussion

Polyphenols are an important ingredient in the diet, and approximately 50% of the daily intake of polyphenols are phenolic compounds [29]. As CGA is one of the most abundant phenolic compounds in the human diet, its anti-inflammatory, antioxidant and various biological activities have been widely reported [10–12, 14, 30–32].

Acute pancreatitis is an inflammatory disease with a varied clinical course. Management is usually conservative, with interventional therapy reserved for those with complications [1, 5]. In our study, we shed light upon the potential role of anti-inflammatory properties of CGA in the treatment of AP using the L-arginine animal model. The benefits of using this model are obvious: it is inexpensive, technically simple to execute, and only requires *ip* injections. The induction method is relatively noninvasive and does not require anesthesia or surgery. Moreover, it accurately reproduces the majority of laboratory and morphological features of human AP. One distinction between human and L-arginine-induced AP is that human disease is typically patchy in distribution, whereas L-arginine-induced AP is relatively homogeneous (more so the case in rats). Unfortunately, the dose of L-arginine that causes AP is very close to the toxic dose, and the severity of the disease is difficult to control (particularly in mice) and administration of excessive doses of L-arginine can cause systemic toxicity and death of animals which might be a limitation of the study [33, 34].

The results of our experiments suggested that CGA may display a highly significant anti-inflammatory activity in a well-established mouse model of L-arginine-induced AP as evidenced by reduction of MPO activity and—noteworthy—histopathological changes (H&E staining, Masson’s trichrome staining, IHC/anti-coll-1, IHC/anti-MMP-1). Microscopically, AP is presented as an inflammatory tissue reaction to functional and/or structural acinar cell damage and rarely duct cell necrosis. Here, morphological assessment showed that the treatment with CGA after the induction of AP reduced inflammatory infiltrates in the periacinar area as well as there was a minimal atrophy of acinar cells. Moreover, lowered amylase activity in the pancreas of the AP group treated with CGA emphasized the positive effect of this compound on murine pancreas.

Our study, along with previously published reports, shows that CGA may become a new therapeutic or an attractive supplement in the treatment of AP. Moreover, our data show that CGA may have therapeutic activity and may be used in patients regardless of the current stage of the disease. Our observations elegantly complete the data obtained by Ohkawara et al*.* [35] who evaluated the protective effect of CGA on the inflammatory damage of pancreas in mice with L-arginine-induced AP. In their study mice were *ip* injected with CGA (20 and 40 mg/kg) 1 h before administration of L-arginine. This pretreatment significantly decreased the histological severity of pancreatitis. Moreover, administration of CGA decreased the levels of pancreatic enzyme activity. Interestingly, CGA reduced the serum and pancreatic levels of macrophage migration inhibitory factor (MIF)—a pro-inflammatory cytokine, which is implicated in cancer (i.e., it is found in many human cancer and cancer-prone inflammatory diseases, including chronic pancreatitis and pancreatic cancer) and may be characterized as critical mediator of severe AP [35, 38, 39]. Even though our study might be seen as similar to the research of Ohkawara et al*.* [35], it needs to be underlined here that we tried to evidence the anti-inflammatory, rather than preventive or protective, effect of CGA.

A limitation of the study is the lack of additional experimental group: mice with CGA alone. However, the chosen dosage of CGA is 20 mg/kg BW twice daily which is about 1200 mg/60 kg BW for the human being. A clinical study employing this dose has already been conducted (https://clinicaltrials.gov/ct2/show/study/NCT02621060, https://clinicaltrials.gov/ct2/show/NCT02136342) and there was no evidence for this dose being toxic. Second, we have designed our study based on the published literature [35] in which similar doses were used. Furthermore, previous studies have shown that CGA has an anti-inflammatory effect under inflammatory conditions such as liver injury in mice [36]. In animal models of IBD, administration of CGA significantly reduced the severity of colitis induced by dextran sulfate sodium in C57BL/6 mice [37].

We are also aware of the limitations of the study resulting from the use of an animal model due to anatomical and physiological differences. On the one hand, human studies are inherently limited to small samples at irregular and often unpredictable intervals, and therefore animal models have largely subdued the research field, with the mouse model being the most used [40]. On the other hand, in human pancreas the bile and pancreatic ducts are separate ducts that normally form a very short common channel in the duodenal wall, whereas those of the rat form a long common channel with the bile duct also serving as a conduit of pancreatic juice. Further, the mouse pancreas is not a well-defined solid organ like in humans, but rather is a diffusely distributed soft tissue while the human pancreas is a single solid organ abutting the duodenal wall. Notwithstanding, there are also some common features between mouse and human pancreas. First, the pancreatic duct drains into the duodenum. Second, cellular components such as acinar, ductal, stellate, and endocrine cells are similar. Third, the mouse pancreas also performs exocrine and endocrine functions [41]. Therefore, another limitation of the study are the morphological differences between human and mouse pancreatic tissue which appear to be numerous, implying that human tissue should be included in basic and applied research [40].

Finally, though most research on CGA shows that it is a fairly safe dietary supplement, one study showed that the use of CGA in combination with a high-fat diet not only does not contribute to weight loss, but also does not protect experimental animals against the adverse metabolic consequences of such a diet [30]. Moreover, the absorption and bioavailability of CGA are still debated due to the large interindividual variations in its utilization, metabolism, and excretion evidenced in both basic and clinical studies [17, 42, 43]. Finally, further research into the therapeutic effects of CGA requires an assessment of its long-term effectiveness and possible side effects of the therapy. Therefore, large and well-designed randomized trials are needed to fully characterize and then exploit the potential of CGA. Nevertheless, the results we obtained along with previous papers suggest that CGA may become an effective and safe drug (proposed dosage: 1200 mg/60 kg BW for the human being) or supplement successfully used in the prevention and treatment of AP.

## Abbreviations

anti-coll-1: Anti-collagen 1
AP: Acute pancreatitis
BW: Body weight
CGA: Chlorogenic acid
COX-2: Cyclooxygenase-2
DMSO: Dimethyl sulfoxide
ECM: Extracellular matrix
HTAB: Hexadecyltrimethylammonium bromide
IL: Interleukin
iNOS: Inducible nitric oxide synthase
*ip*: Intraperitoneal
MPO: Myeloperoxidase
MMPs: Matrix metalloproteinases
MMP-1: Matrix metalloproteinase 1 (collagenase)
NF-κB: Nuclear factor kappa-light-chain-enhancer of activated B cells
OD: Optical density
*po*: Oral gavage
ROS: Reactive oxygen species
SIRS: Systemic inflammatory response syndrome
TNF-*α*: Tumor necrosis factor α
U: Units
